# Supplementary material for: Comparative Analysis of the Nutritional and Sensory Profiles of Commercial Processed Meat Products Made from Beef and Plant-Based Protein
Source: Nutrients. 2025 May 23;17(11):1771. doi: 10.3390/nu17111771 (PMC12157773; doi:10.3390/nu17111771)
Supplement: Supplementary file 1 [file nutrients-17-01771-s001.zip › nutrients-3640885-supplementary.pdf]

Table S1. Nutrient composition and sensory characteristics of the commercial processed meat products from beef and plant-based meat analogs

Traits	BP-1	BP-2	BP-3	PP-1	PP-2	PP-3	SEM
Proximate composition (%)							
Moisture	57.23^{a}	53.72^{bc}	51.73^{c}	51.72^{c}	55.57^{ab}	55.57^{ab}	0.432
Crude protein	13.68^{b}	11.83^{cd}	13.60^{b}	12.44^{bc}	10.28^{d}	18.24^{a}	0.347
Crude lipid	19.25^{c}	22.48^{b}	25.06^{a}	15.01^{d}	13.97^{d}	4.44^{e}	0.278
Carbohydrate	8.42^{c}	10.88^{b}	8.10^{c}	18.30^{a}	18.79^{a}	19.13^{a}	0.372
Crude ash	1.42^{b}	1.10^{c}	1.51^{b}	2.52^{a}	1.39^{bc}	2.62^{a}	0.066
Total calories (kcal/100g)	261.66^{c}	293.15^{b}	312.36^{a}	258.09^{c}	242.00^{d}	189.48^{e}	2.067
Cholesterol (mg/100 g)	48.51^{b}	47.13^{b}	58.36^{a}	N.D.	N.D.	N.D.	1.367
Mineral contents (mg/100 g)							
Ca	12.35^{f}	58.57^{c}	14.70^{e}	90.75^{b}	39.45^{d}	96.68^{a}	0.246
Fe	1.50^{c}	1.00^{f}	1.10^{d}	1.60^{b}	1.03^{e}	2.05^{a}	0.007
K	187.73^{d}	170.25^{e}	240.43^{b}	324.85^{a}	101.00^{f}	206.17^{c}	0.489
Mg	17.62^{e}	19.65^{d}	20.65^{c}	53.88^{a}	19.37^{d}	44.12^{b}	0.118
Na	261.55^{e}	242.18^{f}	311.17^{d}	552.77^{b}	472.70^{c}	603.68^{a}	1.184
Zn	3.15^{b}	2.85^{c}	3.25^{a}	1.25^{d}	1.25^{d}	0.82^{e}	0.007
P	116.03^{f}	220.65^{b}	217.18^{a}	288.43^{a}	146.98^{e}	205.65^{d}	0.474
Cu	0.15^{b}	0.75^{a}	0.05^{d}	0.10^{c}	0.10^{c}	0.10^{c}	0.000
Al	N.D.	N.D.	0.52^{b}	0.10^{d}	0.33^{c}	3.03^{a}	0.014
Sugar contents (g/100 g)							
Fructose	0.15^{b}	0.14^{bc}	0.02^{d}	0.08^{cd}	0.52^{a}	0.10^{bc}	0.014
Glucose	0.26^{c}	0.28^{c}	0.16^{c}	0.57^{b}	2.88^{a}	0.46^{b}	0.037
Sucrose	2.46^{c}	2.06^{c}	1.94^{c}	10.64^{a}	3.30^{c}	6.55^{b}	0.580
Maltose	0.30^{b}	0.42^{ab}	0.85^{a}	N.D.	N.D.	N.D.	0.108
Lactose	N.D.	N.D.	N.D.	N.D.	N.D.	N.D.	-
Total	3.16^{c}	2.89^{c}	2.97^{c}	11.28^{a}	6.70^{b}	7.12^{b}	0.582
Fatty acid composition (%)							
C8:0 (Caprylic acid)	0.20^{c}	0.21^{c}	0.20^{c}	4.93^{b}	5.23^{ab}	5.39^{a}	0.085
C10:0 (Capric acid)	0.12^{c}	0.12^{c}	0.12^{c}	3.51^{a}	3.23^{b}	3.31^{b}	0.023
C12:0 (Lauric acid)	1.69^{b}	1.74^{b}	1.79^{b}	9.94^{a}	10.28^{a}	10.44^{a}	0.151
C14:0 (Myristic acid)	2.21^{b}	2.23^{b}	2.23^{b}	6.93^{a}	6.99^{a}	6.90^{a}	0.071
C16:0 (Palmitic acid)	25.31^{a}	25.34^{a}	25.96^{a}	8.43^{b}	7.91^{b}	8.24^{b}	0.149
C16:1 (Palmitoleic acid)	2.60^{a}	2.58^{ab}	2.45^{b}	0.33^{c}	0.32^{c}	0.32^{c}	0.029
C18:0 (Stearic acid)	17.50^{a}	17.32^{a}	16.20^{a}	6.14^{b}	6.42^{b}	6.24^{b}	0.368
C18:1n9 (Oleic acid)	38.72^{a}	38.64^{a}	39.64^{a}	31.01^{b}	31.27^{b}	30.24^{b}	0.553
C18:1n7 (Vaccenic Acid)	1.39	1.41	1.33	N.D.	N.D.	N.D.	0.018
C18:2n6 (Linoleic acid)	6.19^{b}	6.27^{b}	5.98^{b}	17.34^{a}	16.74^{a}	17.25^{a}	0.299
C18:3n3 (α-Linolenic acid)	2.97^{b}	3.02^{b}	3.02^{b}	8.01^{a}	8.25^{a}	8.32^{a}	0.113
C20:0 (Arachidic acid)	0.35^{c}	0.34^{c}	0.33^{c}	1.06^{a}	1.00^{ab}	0.96^{b}	0.015
C20:1n9 (Eicosenoic acid)	0.65^{b}	0.67^{b}	0.66^{b}	1.76^{a}	1.77^{a}	1.78^{a}	0.020
C20:3n3 (Eicosatrienoic acid)	0.06	0.06	0.06	N.D.	N.D.	N.D.	0.001
C20:4n6 (Arachidonic acid)	0.04	0.04	0.04	N.D.	N.D.	N.D.	0.001
C22:0 (Behenic acid)	N.D.	N.D.	N.D.	0.61^{a}	0.58^{b}	0.61^{a}	0.006
SFA	47.38^{a}	47.31^{a}	46.83^{a}	41.54^{b}	41.65^{b}	42.09^{b}	0.490
UFA	52.62^{b}	52.69^{b}	53.17^{b}	58.46^{a}	58.35^{a}	57.91^{a}	0.490
MUFA	43.36^{a}	43.30^{a}	44.08^{a}	33.10^{b}	33.36^{b}	32.34^{b}	0.531
PUFA	9.26^{b}	9.39^{b}	9.09^{b}	25.36^{a}	25.00^{a}	25.57^{a}	0.304
MUFA/SFA	0.92^{a}	0.92^{a}	0.94^{a}	0.80^{b}	0.80^{b}	0.77^{b}	0.021

PUFA/SFA	0.20^{b}	0.20^{b}	0.19^{b}	0.61^{a}	0.60^{a}	0.61^{a}	0.010
Amino acid (% in protein)							
Aspartic acid	9.48^{bc}	8.96^{bc}	9.03^{bc}	12.20^{a}	8.60^{c}	9.75^{b}	0.193
Threonine	3.58^{a}	3.01^{b}	3.07^{b}	2.88^{b}	2.42^{c}	2.88^{b}	0.093
Serine	2.56^{b}	2.50^{b}	2.49^{b}	3.56^{a}	3.47^{a}	3.70^{a}	0.103
Glutamic acid	20.48^{c}	16.88^{d}	16.44^{d}	19.45^{c}	27.14^{a}	25.28^{b}	0.279
Proline	4.62^{c}	7.24^{ab}	6.98^{b}	5.06^{c}	8.01^{a}	7.41^{ab}	0.164
Glycine	5.70^{c}	10.09^{b}	10.72^{a}	4.69^{d}	4.18^{d}	4.25^{d}	0.124
Alanine	6.57^{b}	7.56^{a}	7.86^{a}	5.23^{c}	4.41^{d}	4.43^{d}	0.153
Valine	5.71	5.47	5.41	5.93	5.54	5.46	0.131
Isoleucine	5.05^{ab}	4.56^{b}	4.42^{b}	5.42^{a}	5.02^{ab}	5.03^{ab}	0.151
Leucine	8.53	7.78	7.63	8.51	8.34	8.20	0.197
Tyrosine	1.37	1.57	1.58	1.45	1.40	1.41	0.075
Phenylalanine	5.03^{bc}	4.73^{c}	4.65^{c}	5.85^{a}	5.77^{a}	5.74^{ab}	0.155
Histidine	5.58^{a}	4.54^{b}	4.47^{b}	5.12^{ab}	4.59^{b}	4.65^{b}	0.146
Lysine	9.22^{a}	8.28^{b}	8.21^{b}	6.95^{c}	4.82^{e}	5.30^{d}	0.052
Arginine	6.51^{b}	6.85^{ab}	7.04^{ab}	7.71^{a}	6.27^{b}	6.51^{b}	0.192
Sensory evaluation							
Appearance	7.43^{a}	7.25^{a}	7.58^{a}	7.15^{ab}	6.71^{b}	5.90^{c}	0.123
Color	7.37^{a}	7.26^{ab}	7.50^{a}	7.24^{ab}	6.77^{b}	5.85^{c}	0.127
Off-flavor	2.67^{c}	2.96^{c}	2.77^{c}	3.71^{b}	3.70^{b}	4.84^{a}	0.179
Taste	7.53^{a}	6.42^{b}	7.47^{a}	5.75^{c}	5.48^{c}	3.22^{d}	0.143
Flavor	7.20^{a}	6.29^{b}	7.22^{a}	5.62^{c}	5.34^{c}	3.19^{d}	0.150
Juiciness	5.77^{a}	5.81^{a}	5.88^{a}	5.13^{b}	5.79^{a}	3.75^{c}	0.148
Tenderness	6.05^{bc}	6.38^{ab}	5.85^{bc}	6.70^{a}	6.27^{ab}	5.52^{c}	0.140
Overall acceptability	7.40^{a}	6.62^{b}	7.37^{a}	5.84^{c}	5.46^{c}	3.50^{d}	0.136

$^{a\text{-}f}$ Means within a row with different a superscript differ significantly at $p<0.05$.

BP, beef product; PP, plant-based meat analog product; N.D., not detected; SFA, saturated fatty acids; UFA, unsaturated fatty acids; MUFA, monounsaturated fatty acids; PUFA, polyunsaturated fatty acids; SEM, standard error of the mean.

The content of carbohydrates was calculated as follows: 100 − (Moisture + Protein + Ash + Lipid).

Appearance, color, taste, flavor, overall acceptability (1 = very bad, 9 = very good), off-flavor (1 = very weak, 9 = very strong), juiciness (1 = very dry, 9 = very juicy), and tenderness (1 = very hard, 9 = very tender).
